# Heterogeneity of false reactivity profiles of HIV assays while optimizing national HIV testing algorithms: Findings from a multi-country analysis

**DOI:** 10.1016/j.jcv.2025.105843

**Published:** 2025-10

**Authors:** Manfred Accrombessi, Cheryl Johnson, Alaleh Abadpour, Jean De Dieu Anoubissi, Joseph Fokam, Araz Chiloyan, Iryna Andrianova, Hamakwa Mantina, Agai Kherbino Akec, Fatou Ousmane Sall, Abdelaye Keita, Elizabeth Telan, Adoum Fouda Abderrazzack, Chatté Adawaye, Rose Wafula, Stephen Ayisi-Addo, Monkoe S. Leqheka, Jacob Lusekelo Mwambeta, Jeremie Muwonga Masidi, Dramane Kania, Anita Sands, Rachel Baggaley, Jean-François Etard, Céline Lastrucci

**Affiliations:** a Epigreen, Paris, France; b World Health Organization, Global HIV Hepatitis, and STI Programmes, Geneva, Switzerland; c Central Technical Group, National AIDS Control Committee, Yaounde, Cameroon; d Chantal BIYA International Reference Centre for HIV/AIDS Prevention and Management, Faculty of Medicine and Biomedical Sciences of the University of Yaoundé, Cameroon; e National Center of Infectious Diseases, Ministry of Health, Republic of Armenia; f HIV/AIDS Reference Laboratory, Public Health Center, Ministry of Health, Ukraine; g Ministry of Health, Zambia; h National HIV/Hepatitis/STIs Program, Ministry of Health, South Sudan; i Institut National de Santé Publique, Ministry of Health, Mali; j National Reference Laboratory, Ministry of Health, Philippines; k Ministry of Public Health/University of N’Djamena, Chad; l National AIDS and STIs Control Program, Ministry of Health, Kenya; m National HIV and AIDS Control Program, Ministry of Health, Ghana; n Laboratory Services, Ministry of Health, Lesotho; o Diagnostic Services, Ministry of Health, Tanzania; p National HIV Control Program, University of Kinshasa, Democratic Republic of the Congo; q Centre Muraz, INSP, Burkina Faso; r WHO, Regulation and Prequalification Department, Switzerland

**Keywords:** HIV RDT, False reactivity, HIV testing strategies and algorithms, Heterogeneity, False positivity

## Abstract

This study highlights the importance of verifying HIV testing algorithms to reduce the risk of misdiagnoses caused by common false reactivity. Between 2020 and 2023, WHO supported 14 countries to assess rates of false reactivity and shared false reactivity across HIV rapid diagnostic tests (RDTs) used in HIV testing services. The study involved 26,278 results from 22 different RDT products, with sample sizes ranging from 100 to 302 results per country. The number of RDT products assessed varied between 4 and 13 per country. False reactivity rates ranged from 0 % to 3.32 %, with one country reporting a high false reactivity rate of over 4 % for one RDT. Five countries have no shared false reactivity between RDTs, while the remaining eight countries shared false reactivity across one to six pairs of RDT products. These findings were used to inform national policy, with more than 90 % of countries introducing new RDT products into their HIV testing algorithm based on these results. The study concludes that rates of false reactivity and shared false reactivity between RDT products vary across countries. Therefore, conducting verification studies is crucial for updating national HIV testing algorithms and ensuring accurate diagnosis while also facilitating the market entry of new HIV testing products.

## Highlights

● HIV verification studies are typically fast and straightforward to conduct when the necessary supplies are readily available on-site.
● False reactivity rates and the extent of shared false reactivity between HIV rapid diagnostic tests (RDTs) differ markedly across countries.
● All 14 countries used the study’s results to update their national HIV testing algorithms. Among countries using RDTs before the study, more than 90 % introduced at least one new test in their HIV testing algorithm based on the findings.

## 1 Introduction

HIV testing services (HTS) are a critical gateway to treatment and prevention, playing a key role in achieving the 95-95-95 targets by 2030 [1]. World Health Organization (WHO) recommends a mixed approach for HTS to maximize impact and efficiency, which includes testing in healthcare facilities and in community settings, as well as network based testing and self-testing [2]. Accurate, timely and affordable HIV diagnosis at the point of care is vital to reach people unaware of their HIV status and ensure rapid initiation of HIV care and prevention services. WHO recommends all countries adopt a 3-test strategy for reliable HIV diagnosis, regardless of national HIV prevalence. This strategy requires three sequential HIV serological assays, including both rapid diagnostic tests (RDTs) and enzyme immunoassays. A HIV-positive diagnosis is provided when three consecutive assays are reactive [3], [4]. In specific settings such as antenatal care and among key populations (men who have sex with men, sex workers, people who inject drugs, trans-and gender diverse people, people in prison), WHO recommends that the first RDT (A1) in this 3-test strategy should detect both HIV and syphilis infections [5]. Each HIV RDT product must achieve minimum performance standards of ≥99 % sensitivity (detecting 99 out of 100 true positive cases) and ≥98 % specificity (correctly identifying 98 out of 100 true negative cases) [6], [7]. To prevent misdiagnosis from poorly selected algorithms [8], WHO recommends verification studies prior to widespread implementation, following the protocols outlined in the latest WHO guidance and resources [7], [9]. The verification study doesn't assess test performance, as all included products have already undergone WHO prequalification with independent performance evaluation. Instead, it aims to evaluate false reactivity (FR) patterns among pre-selected products to construct a three-test testing algorithm using products with minimal or no shared false reactivity.

Several countries have conducted the recommended verification studies to select their national HIV testing algorithms. In this report, we present the results of a multi-country secondary data analysis aimed at evaluating the false reactivity profile of each product and the extent of shared false reactivity across the different products used in these national studies. The results will help align country guidelines and practices with current WHO recommendations, ensuring selected testing algorithms minimize misdiagnosis risk before wider implementation.

## 2 Materials and methods

### 2.1 Verification study design

Laboratory data from verification studies conducted by each participating country was included in the multi-country analysis.

Briefly, a verification study consisted of prospectively collecting between 100 and 230 HIV-negative specimens from individuals attending routine HTS, including antenatal care. Specimens were characterized as HIV-negative using a parallel testing strategy comprising one EIA and one RDT. Positive and discordant specimens are excluded from the panel.

This sample size was calculated to detect a 2–8 % false reactivity rate from products and assess the extent of shared false reactivity between them. Inclusion criteria were i) aged above 18 years; ii) seeking HTS; iii) able to understand the scope of the study and provide written informed consent. Individuals with a known (self-reported) HIV-positive status and/or on antiretroviral therapy (self-reported) were excluded.

Participating countries selected WHO-prequalified products assays (except two products) to include in the study, typically prioritizing 10–12 products based on performance (e.g., sensitivity, specificity), other operational characteristics (e.g. storage conditions, shelf life, ease of use) and pricing. Two separate production lots (denoted as lot A and lot B) of each shortlisted product were tested against the panel of HIV-negative specimens. False reactivity was confirmed if the result on lot A or lot B result were reactive. To reduce bias, the products used to confirm HIV-negative status of the specimen panel were not eligible to be candidate products in the study.

Countries selected their national testing algorithms by choosing products with lowest false reactivity rates and little to no shared false reactivity on the same specimens. Study findings, along with performance and operational criteria for each product, were then used to guide countries in making their final algorithm selection. More details are published elsewhere including the generic WHO protocol [9].

Each verification study was submitted for institutional approval and obtained ethical clearance from the National Health Research Ethics Committee as well as the WHO Ethical Review Board.

### 2.2 Data management and analysis

Fourteen countries who collaborated with WHO agreed to share their anonymous databases for this multi-country analysis. Data consistency was verified through double-checking of all entries by either a data clerk or a co-investigator before sharing.

The country data was merged, cleaned, and analyzed using Stata 16.0 software (Stata Corp, College Station, TX, USA). All data included in this manuscript belongs to the countries. To respect confidentiality and avoid potential use of those results for marketing purposes, countries and product names were anonymized.

Descriptive statistics were used to summarize the data from the verification studies. First, we assessed the number of countries that evaluated each product, as well as the number of common products examined by pairs of countries and displayed this information using a heatmap. We tabulated the false reactivity by product to determine the overall frequency of false reactivity stratified by country. Secondly, we analyzed the shared false reactivity profile by country. We defined it as false cross-reactivity occurring when at least two products showed reactivity on the same specimen.

Lastly, to illustrate the heterogeneity of false and shared false reactivities across countries, we selected countries that tested the same set of products.

To assess the introduction of new RDT products into the national market following the verification studies, we analyzed the proportion of countries that did include at least one new test in their testing algorithms.

## 3 Results

Data from fourteen participating countries were included in the analyses: Burkina Faso, Ghana, Mali, Cameroon, Chad, the Democratic Republic of Congo (DRC), Kenya, Zambia, South Sudan, Lesotho, Ukraine, Armenia, the Philippines, and the United Republic of Tanzania.

**Fig. 1:**
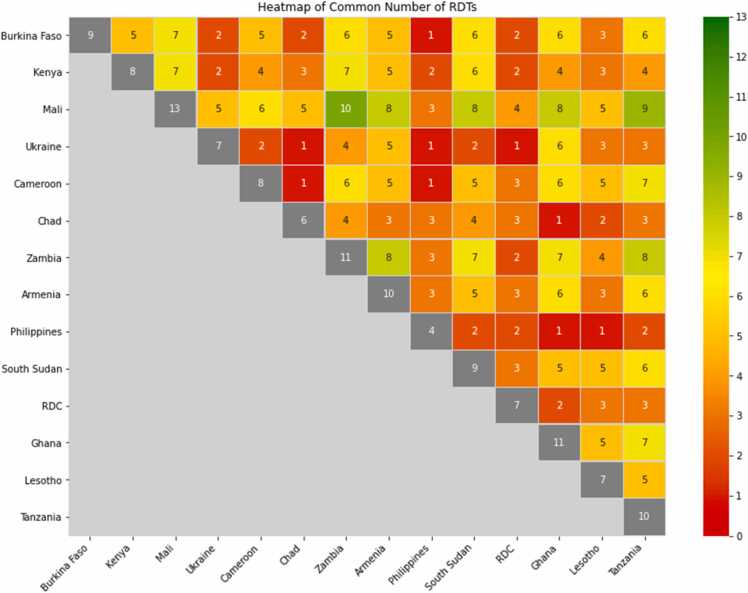
Heatmap showing the number of common products by pairs of countries.

Only products in the form of rapid diagnostic tests (RDTs) were included by the participating countries. The list of the assays and their individual code used in this analysis can be fund in Table 1. The number of products tested varied among countries, ranging from 4 to 13 (median = 10), while the number of countries testing each product ranged from 1 to 12 (median = 6), as shown in Table 2. In total, 22 products were evaluated. Notably, several countries shared the same set of products (see Fig. 1). The number of common products was primarily influenced by the total number of products tested by each country.

**Table 1:** Tests assessed.

Code No	Tests
1	Bioline HIV 1/2 3.0 Abbott Diagnostics Korea Inc
2	Bioline HIV/Syphilis Abbott Diagnostics Korea
3	Determine HIV Early Detec Abbott Diagnostics Medical Co. Ltd
4	Determine HIV1/2 Abbott Diagnostics Medical Co. Ltd
5	Diagnostic kit for HIV (1 + 2) antibody (colloidal gold) V2 Shanghai Kehua Bio-engineering Co., Ltd
6	EXACTO-PRO HIV test BioSynex Esay Diagnostics for life
7	First response HIV 1–2. O Card test (version 2.0) Premier Medical Corporation
8	First response HIV1 + 2/Syphilis Combo card Test Premier Medical corporation
9	Genie Fast HIV1/2 Bio-Rad
10	HIV 1/2 STAT PAK Chembio Diagnostic Systems Inc
11	INSTI HIV-1/HIV-2 Antibody Test BioLytical Laboratories Inc.
12	MERISCREEN HIV 1–2 WB Meril Diagnostics Pvt. Ltd.
13	ONE STEP Anti‐HIV (1&2) Test InTec PRODUCTS, INC
14	ONE STEP HIV1/2 Whole Blood/Serum/Plasma Test Guangzhou Wondfo Biotech Co., Ltd
15	OraQuick HIV1/2 test Orasure Technologies
16	QUICK PROFILE HIV 1/2
17	Rapid Test for Antibody to HIV (Colloidal Gold Device) Beijing Wantai Biological Pharmacy Enterprise Co.
18	STANDARD Q HIV 1/2 Ab 3-Line Test SD Biosensor, Inc
19	STANDARD Q HIV/Syphilis Combo Test SD Biosensor Inc
20	SURE CHECK HIV 1/2 ASSAY Diagnostic Chembio Systems
21	TrinScreen HIV Trinity Biotech Manufacturing Ltd.
22	Uni-Gold HIV Trinity Biotech Manufacturing Ltd.

**Table 2:** General verification studies characteristics, 14 countries.

Characteristics	Mean	Median (IQR)	Range
Number of specimens per country	233.8 (±46.5)	230 (200–252)	100–302
Number test events/points per country	4284.2 (±1433.4)	4800 (4000–5040)	1200–5995
Number products assessed per country	9.71 (±2.2)	10 (9–13)	4–13
Number of countries that examined the same product	5.45 (±3.3)	6 (3–8)	1–12

Table 3 presents the overall false reactivity for each product across all countries, ranked by the frequency of false reactivity. Six products had a false reactivity of 1 % or higher, with two exceeding the 2 % threshold, that is 98 % specificity. Overall, 16 of the 22 products tested (72 %) exhibited a false reactivity below 1 %, and three products showed no false reactivity at all. Results for the last two products (RDT 15 and 20) were based on the smallest sample size, 225 and 245, respectively. “RDT 4″ was the most extensively studied product, evaluated in 11 countries with 2619 results included in the analysis, and it had a false reactivity of 0.95 %.

**Table 3:** Overall false reactivity (FR) across all countries, per RDT, sorted by frequency of FR.

RDT product	Nb countries using	No. of negative specimens	No. of reactive specimens	False reactive (%)	False reactive min (%)	False reactive max (%)
RDT 17	4	782	26	3.32	0.50	7.54
RDT 6	1	245	5	2.04	2.04	2.04
RDT 9	3	629	9	1.43	0.00	2.32
RDT 21	2	533	7	1.31	0.43	2.01
RDT 5	3	681	8	1.17	0.40	2.62
RDT 10	8	1788	15	1.03	0.00	3.57
RDT 16	1	300	3	1.00	1.00	1.00
RDT 4	12	2619	25	0.95	0	2.63
RDT 2	10	2199	15	0.68	0	3.51
RDT 3	6	1271	8	0.62	0.00	2.50
RDT 19	9	1969	11	0.59	0.00	4.00
RDT 18	7	1638	10	0.56	0.00	1.98
RDT 14	8	1898	10	0.53	0.00	1.74
RDT 13	6	1281	7	0.47	0.00	1.22
RDT 22	6	1203	5	0.46	0.00	2.50
RDT 11	4	763	2	0.31	0.00	1.00
RDT 8	8	1714	5	0.29	0.00	1.66
RDT 7	9	1875	3	0.17	0.00	1.50
RDT 12	3	684	1	0.15	0.00	0.44
RDT 20	1	245	0	0.00	0.0	0.00
RDT 1	8	1736	0	0.00	0.0	0.00
RDT 15	1	225	0	0.00	0.0	0.00

**Fig. 2:**
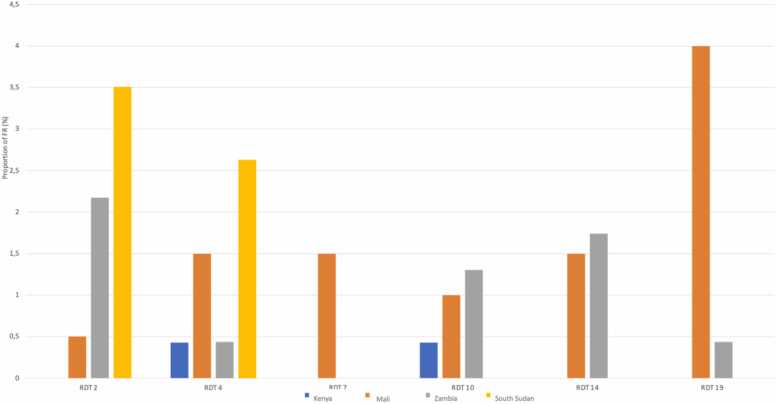

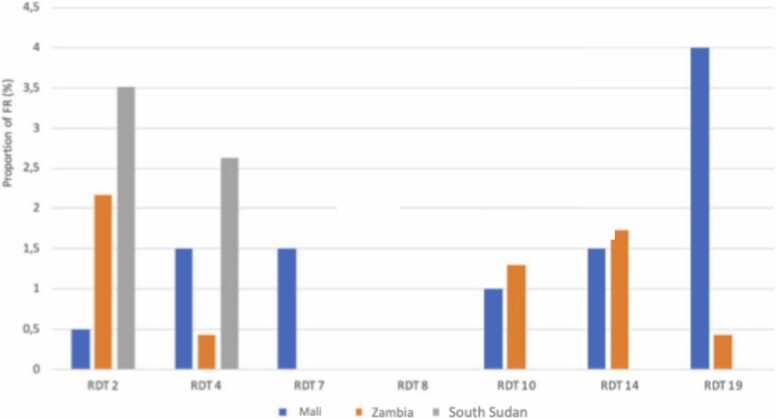
(a). False reactivity proportion by product and country (4 countries, six common products). (b). False reactivity proportion by product and country (3 countries, seven common products).

We observed significant heterogeneity in false reactivity across countries when we restricted the analysis to countries using the same products (see Fig. 2a and b). Notably, in Fig. 2a, six products displayed false reactivity in MaliMali, while fewer products showed false reactivity in KenyaKenya and SouthSouth Sudan. Similar heterogeneity can be observed in Fig. 2b on another set of countries and products. This underscores the discrepancies between countries, where false reactivity rates can vary considerably.

Table 4 displays the number of pairs of products sharing false reactivity, regardless of the country. “RDT 2” and “RDT 4” were the most frequently involved products in shared false reactivity, with nine pairs identified; notably, these were also the two most examined products. Eight products had no shared false reactivity (indicated in blue), and among these, three did not yield any false results (RDTs 1, 15, and 20). The last two products were only tested in one country.

**Table 4:** Matrix of shared false reactivity for HIV rapid diagnostic tests.

Table 5, Table 6 illustrate the heterogeneity in shared false reactivity profile between countries for specific pairs of products, showcasing two countries that evaluated same three pairs of products, the share false reactivity differs: RDT 2–14, RDT 4–18 and RDT 7–19 cross-reacted on same negative specimen in MaliMali but not in ZambiaZambia. These results, once again, highlight the profile heterogeneity between countries, complicating the use of individual country findings by other countries.

**Table 5:** Shared FR for three common pairs of products with Mali.

**Table 6:** Shared FR for three common pairs of products with Zambia.

As illustrated in Table 7, of the 14 countries, 64 % (9/14) identified at least one shared false reactivity. Among the 12 countries already having an RDT-based testing algorithm prior to the study, 11 decided to change at least one product in their algorithm, and 50 % (6/12) decided to change the product used in the first (A1) position. This illustrates the opportunity for products included in verification studies to be taken up by national program.

**Table 7:** National algorithm decision making process outcomes by country.

Country	Shared FR identified	A1 changed	A2 changed	A3 changed
Burkina Faso	Yes	Yes	Yes	Yes
Kenya	No	Yes	Yes	Yes
Mali	Yes	Yes	No	NA (no A3 before)
Ukraine	No	NA: RDTs not used before		
Cameroon	Yes	Yes	Yes	No
Chad	No	No	Yes	Yes
Zambia	Yes	Yes	No	NA (no A3 before)
Armenia	Yes	NA: RDTs not used before		
Philippines	Yes	No	No	Yes
SouthSouth	Yes	No	Yes	NA (no A3 before)
DRC	Yes	No	No	Yes
Ghana	No	Yes	No	No
Lesotho	No	No	No	Yes
Tanzania	Yes	No	No	NA (no A3 before)

## 4 Discussion

WHO recommends countries review their testing algorithms at regular intervals to respond to emerging trends or new products available on the market (including locally manufactured products) [7]. Our study directly demonstrates how studies that verify national HIV testing algorithms can play a pivotal role in informing national stakeholders and driving evidence-based product selection. The value of these studies lies in their practicality and ability to quickly produce actionable evidence, with limited resources, that supports the optimization of testing algorithms, ensuring they are adapted to the local epidemiological and operational contexts.

Eleven of the 12 countries already using RDT-based testing algorithms before the study took the opportunity of those verification studies to include new products (including new A1 for 50 %) into their national HIV testing algorithms. Reasons for including new tests into national testing algorithms were context specific. Each country identified a combination of performance and operational criteria and weighted those criteria according to their specific context. Engaging a multidisciplinary working group including relevant national programs, procurement agencies, testing providers, national reference laboratories, technical and financial partners to analyze data was found to be useful to ensure consensus and buy-in. In addition to performance criteria (including false reactivity and shared false reactivity), ease of use, shelf life and storage conditions were the frequent operational criteria used to select country specific testing algorithms along with cost. This suggests that verification studies can support the global effort to drive equitable access to affordable, locally produced, and quality-assured products by promoting the introduction of new products into the national guidelines [10], [11]. Verification studies align well with the need to provide countries with evidence to refine their testing algorithms based on local data considering product performance, false reactivity analysis, and operational factors. By implementing these verification studies at the national level, some countries have already successfully adapted their HIV testing algorithms by replacing previously used products with new alternatives. These early adopters demonstrate how verification studies can directly influence national health policies by enabling the entry of new, quality-assured products into HTS.

In this multi-country analysis covering 14 countries worldwide, we observed substantial heterogeneity in false reactivity rates. Some products displayed no false reactivity in one country but a high false reactivity rate in another country, and we observed the same for shared false reactivity. These findings further underscore the need for locally conducted verification studies, as false reactivity rates and shared false reactivity may vary significantly between countries, implicating the generalizability of specific country results by another country. In this analysis, we did not identify any regional or product pattern that could be used by another country than the one performing the verification study. As Member States continue to conduct verification studies, WHO is planning to update multi-country analysis as additional study results become available to investigate any common pattern that could be used in the country where it appears complicated to implement the study.

Although the studies are designed to be quick and practical, this requires countries to make available sufficient supplies of candidate products. Weak procurement systems for these relatively small quantities of candidate products meant that not all countries were able to implement at the same speed. Several countries experienced delays in study implementation due to delayed arrival of products, particularly those not already in widespread use domestically. These delays can impede the speed at which countries can review/update their testing algorithm and need to be addressed by improving the efficiency of procurement processes and ensuring manufacturers are sensitized on the need to ship small quantities for the study. Where needed, mechanisms such as the WHO collaborative registration procedure for IVDs (CRP) can facilitate access to products that were not previously registered by relying on WHO prequalification to accelerate national registration and support algorithm scale up [12].

The choice of national HIV testing algorithms is only the beginning of a broader algorithm transition process. Some countries have experienced significant delays between the end of the verification study and l implementation of renewed testing algorithms. To accelerate this process, countries are encouraged to develop comprehensive algorithm transition plans which include the verification study and a national scale up plan. This scale up plan should include updating the supportive tools (standard operating procedures, bench aids, training plans, demand creation materials, data collection and management, etc.), national registration for new products, national quantification, updating quality management systems (QMS). To familiarize testing providers and clients with the new algorithms it is also suggested to include a small-scale implementation phase as part of the national scale up plan. To support this transition process, it has been found very important by countries to set up a multi-sector team (laboratory specialists, HIV national programs, procurement/supply responsible, financial and technical partners, beneficiaries, etc.) to ensure efficient coordination.

Additionally, to ensure continuous quality of testing following national scale up, irrespective of where, how and by whom testing is provided, HTS must implement QMS in all testing sites, including non-laboratory testing sites following the latest available guidance and tools [13], [14], [15], [16].

Despite the importance of these verification studies, the current research has several limitations. First, due to the big volume of specimen required to run all product, the study did not use capillary whole blood specimens. The use of such specimens would have required multiple finger pricks per patient, potentially reducing the acceptability of the study among participants. Second, the study’s primary focus was on identifying products with high specificity that do not share false reactivity on negative specimens. As a result, it does not investigate the causes of false reactive results, particularly within different population groups. The limited collection of sociodemographic data further restricts our understanding of how these factors may influence false reactivity rates. Lastly, while several products were verified in multiple countries, the number of products shared between countries remains limited, and this may affect the generalizability of the results. Future studies should consider a more extensive cross-country comparison with a larger pool of shared products to provide even more robust insights into test performance across different regions. As Member States continue to conduct verification studies, WHO is planning to update this multi-country analysis when additional study results become available.

## CRediT authorship contribution statement

**Chatté Adawaye:** Writing – review & editing. **Alaleh Abadpour:** Writing – review & editing. **Rose Wafula:** Writing – review & editing. **Jean De Dieu Anoubissi:** Writing – review & editing. **Elizabeth Telan:** Writing – review & editing. **Adoum Fouda Abderrazzack:** Writing – review & editing. **Cheryl Johnson:** Writing – review & editing, Supervision. **Jacob Lusekelo Mwambeta:** Writing – review & editing. **Iryna Andrianova:** Writing – review & editing. **Jeremie Muwonga Masidi:** Writing – review & editing. **Hamakwa Mantina:** Writing – review & editing. **Stephen Ayisi-Addo:** Writing – review & editing. **Joseph Fokam:** Writing – review & editing. **Monkoe S. Leqheka:** Writing – review & editing. **Araz Chiloyan:** Writing – review & editing. **Rachel Baggaley:** Writing – review & editing, Project administration. **Abdelaye Keita:** Writing – review & editing. **Jean-François Etard:** Writing – original draft, Methodology, Formal analysis. **Manfred Accrombessi:** Writing – original draft, Methodology, Formal analysis, Data curation. **Dramane Kania:** Writing – review & editing. **Agai Kherbino Akec:** Writing – review & editing. **Anita Sands:** Writing – review & editing, Methodology. **Fatou Ousmane Sall:** Writing – review & editing. **Céline Lastrucci:** Writing – review & editing, Validation, Supervision, Data curation, Conceptualization.

## Funding

WHO receives grants from 10.13039/100000200USAID, the 10.13039/100004417Global Fund, the 10.13039/100000865Bill and Melinda Gates Foundation, 10.13039/100000030CDC, and Unitaid to fund activities related to HIV testing services. This work was supported, in whole or in part, by the 10.13039/100000865Gates Foundation INV-053439. The conclusions and opinions expressed in this work are those of the author(s) alone and shall not be attributed to the Foundation. Under the grant conditions of the Foundation, a Creative Commons Attribution 4.0 License has already been assigned to the Author Accepted Manuscript version that might arise from this submission. Please note works submitted as a preprint have not undergone a peer review process.

## Declaration of Competing Interest

The authors declare that they have no known competing financial interests or personal relationships that could have appeared to influence the work reported in this paper. WHO received grants from Global Fund, USAID, BMGF and Unitaid related to HIV testing services. The manuscript reflects the views of the authors, not the official position of the World Health Organization and funding agencies.

## Data Availability

The datasets generated and/or analyzed during the present study are available on reasonable request from/to the corresponding authors. However, the individual brand names of tests and countries are anonymized due to study and reporting requirements.
